# Association between renin and atherosclerotic burden in subjects with and without type 2 diabetes

**DOI:** 10.1186/s12872-016-0346-8

**Published:** 2016-09-05

**Authors:** Isabel Gonçalves, Andreas Edsfeldt, Helen M. Colhoun, Angela C. Shore, Carlo Palombo, Andrea Natali, Gunilla Nordin Fredrikson, Harry Björkbacka, Maria Wigren, Eva Bengtsson, Gerd Östling, Kunihiko Aizawa, Francesco Casanova, Margaretha Persson, Kim Gooding, Phil Gates, Faisel Khan, Helen C. Looker, Fiona Adams, Jill Belch, Silvia Pinnola, Elena Venturi, Michaela Kozakova, Li-Ming Gan, Volker Schnecke, Jan Nilsson, on behalf of the SUMMIT consortium

**Affiliations:** 1 Department of Clinical Sciences Malmö, Lund University, Malmö, Sweden; 2 Medical Research Institute, University of Dundee, Dundee, UK; 3 Diabetes and Vascular Medicine, NIHR Exeter Clinical Research Facility and University of Exeter Medical School, Exeter, UK; 4 Department of Surgical, Medical, Molecular Pathology, and Critical Area Medicine, Pisa, Italy; 5 Department of Clinical and Experimental Medicine, University of Pisa, Pisa, Italy; 6 AstraZeneca, Cardiovascular and Metabolic Diseases, Mölndal, Sweden

**Keywords:** Renin, Type 2 diabetes, Atherosclerosis, Arterial stiffness, Endothelial dysfunction

## Abstract

**Background:**

Activation of the renin-angiotensin-aldosterone-system (RAAS) has been proposed to contribute to development of vascular complications in type 2 diabetes (T2D). The aim of the present study was to determine if plasma renin levels are associated with the severity of vascular changes in subjects with and without T2D.

**Methods:**

Renin was analyzed by the Proximity Extension Assay in subjects with (*n* = 985) and without (*n* = 515) T2D participating in the SUMMIT (SUrrogate markers for Micro- and Macro-vascular hard endpoints for Innovative diabetes Tools) study and in 205 carotid endarterectomy patients. Vascular changes were assessed by determining ankle-brachial pressure index (ABPI), carotid intima-media thickness (IMT), carotid plaque area, pulse wave velocity (PWV) and the reactivity hyperemia index (RHI).

**Results:**

Plasma renin was elevated in subjects with T2D and demonstrated risk factor-independent association with prevalent cardiovascular disease both in subjects with and without T2D. Renin levels increased with age, body mass index, HbA1c and correlated inversely with HDL. Subjects with T2D had more severe carotid disease, increased arterial stiffness, and impaired endothelial function. Risk factor-independent associations between renin and APBI, bulb IMT, carotid plaque area were observed in both T2D and non-T2D subjects. These associations were independent of treatment with RAAS inhibitors. Only weak associations existed between plasma renin and the expression of pro-inflammatory and fibrous components in plaques from 205 endarterectomy patients.

**Conclusions:**

Our findings provide clinical evidence for associations between systemic RAAS activation and atherosclerotic burden and suggest that this association is of particular importance in T2D.

## Background

Activation of the renin-angiotensin-aldosterone-system (RAAS) has been implicated in the development of vascular complications in type 2 diabetes (T2D) [1, 2]. Approximately 75 % of subjects with T2D have hypertension [3]. Factors contributing to raising blood pressure in T2D include elevated production of angiotensinogen in abdominal fat and hyperinsulinemia-dependent activation of the sympathetic nervous system stimulating renin expression [4, 5]. Renin is the key activator of RAAS [6, 7]. It is primarily produced by the juxtaglomerular apparatus in the afferent arterioles of the kidney and functions by hydrolyzing angiotensinogen into angiotensin I. Angiotensin I is subsequently cleaved by angiotensin-converting enzyme (ACE) to generate angiotensin II, a powerful vasoconstrictor that increases blood pressure [7]. However, angiotensin II has also been reported to have several other biological effects including stimulation of smooth muscle cell proliferation and hypertrophy, oxidative stress, as well as the release of pro-inflammatory cytokines and pro-fibrotic factors that may contribute to the development of macrovascular complications in diabetes also in other ways beyond blood pressure [7–10]. Accordingly, blockade of angiotensin II receptors attenuates the development of atherosclerosis in apolipoprotein E knockout mice with streptozotocin-induced diabetes [11, 12]. However, stimulation of smooth muscle cell proliferation and extracellular matrix synthesis may also help stabilize vulnerable atherosclerotic plaques [13] suggesting that activation RAAS may have both detrimental and beneficial effects on cardiovascular disease in T2D. Intervention studies using ACE inhibitors or angiotensin receptor blockers (ARBs) in patients with diabetes have demonstrated a reduction of cardiovascular events [14–16], but it remains to be fully understood to what extent this involves effects on the vasculature that are unrelated to the effects of RAAS on the blood pressure.

To further explore the relation between activation of RAAS and vascular complications in diabetes we analyzed the association between plasma renin levels and markers of atherosclerosis, arterial stiffness and endothelial dysfunction in 1500 subjects with and without T2D matched for age, gender and prevalence of CVD participating in the SUMMIT (SUrrogate markers for Micro- and Macro-vascular hard endpoints for Innovative diabetes Tools) study. Furthermore to determine if plasma levels of renin were related with atherosclerotic plaque phenotype we analyzed their association with markers of inflammation and fibrous components in 205 carotid plaques obtained at endarterectomy.

## Methods

### Study populations

The SUrrogate markers for Micro- and Macro-vascular hard endpoints for Innovative diabetes Tools (SUMMIT) study cohort consisted of 4 groups; (1) subjects with T2D and clinically manifest CVD, (2) subjects with T2D but without clinical signs of CVD, (3) subjects with CVD but no diabetes and (4) subjects without both CVD and diabetes recruited from existing population cohorts and hospital registers at the university hospitals in Malmö (Sweden), Pisa (Italy), Dundee and Exeter (UK) between December 2010 and April 2013 [17]. Diabetes was defined by current or previous evidence of hyperglycemia (according to WHO 1998 criteria; fasting plasma glucose >7.0 mmol/l or 2-h plasma glucose >11.1 mmol/l, or both) or by current medication with insulin, sulphonylureas, metformin or other anti-diabetic drugs. A clinical history of CVD included a previous diagnosis in the clinical record of non-fatal acute myocardial infarction (MI), hospitalized unstable angina, resuscitated cardiac arrest, any coronary revascularization procedure, non-fatal stroke, transient ischemic attack confirmed by a specialist, lower extremities arterial disease defined as Ankle Brachial Pressure Index (ABPI) <0.9 with intermittent claudication or prior corrective surgery, angioplasty or above ankle amputation. T2D with and without CVD were matched at each center for gender, age (±5 years) and duration of diabetes (±5 years). Subjects without T2D were matched for gender and age (±5 years) at each center. Subjects with CVD with or without T2D were matched for CVD type. Exclusion criteria included renal replacement therapy, malignancy requiring active treatment, end-stage renal disease, any chronic inflammatory disease on therapy, previous bilateral carotid artery invasive interventions or age <40 years. Demographics, clinical characteristics including medication, physical and laboratory examinations were obtained according to a pre-defined study protocol at all 4 participating centers.

### Carotid ultrasound, endothelial function, arterial stiffness and ankle brachial pressure index measurements

Carotid intima media thickness (IMT) was measured both in common carotid artery (CCA) and in the bulb as previously described [17]. To calculate carotid plaque area the proximal-distal boundaries of a plaque were set where the echo of the intima began to diverge from the adventitia echo forming a focal thickening of the intima-media-complex. The plaque area was assessed by outlining the contours of the plaque using the trace function on the ultrasound machine. The plaque area represents the sum of all plaques detected in the carotid artery and the values shown in this study represent the mean of the left and right carotid arteries.

Endothelial function was measured using EndoPat (Itamar Medical, Caesarea Ind. Park, Israel) to estimate the endothelium-dependent vasodilation following post-ischemic hyperemia [17].

The reactive hyperemia index (RHI) was calculated as a post-occlusion to pre-occlusion ratio of the signal amplitudes. Thirty-one subjects were excluded from the RHI analysis due to incomplete occlusion (brachial pulses from the occluded arm were visible during occlusion, despite an increase of the pressure of the cuff to the maximum level of 300 mmHg) or time of occlusion was > or < 5 mins.

Arterial stiffness was assessed by calculating pulse wave velocity (PWV) using a Sphygmocor device (Atcor Medical, Australia). The carotid and femoral pulses were captured. PWV (m/s) was automatically calculated as the measured distance divided by the differences in time between the R wave of the ECG to the foot of the carotid and femoral pulse curves as previously described [17].

The ankle brachial pressure index (ABPI) was calculated as the ratio between the highest systolic blood pressure values from each foot respectively and the blood pressure from the arm giving the highest value. Values given represent the mean of the left and right ABPI.

### Carotid endarterectomy patients and analyses of plaque tissue

Two hundred and five human carotid plaques were collected at carotid endarterectomy. The indications for surgery were plaques associated with ipsilateral symptoms (transitory ischemic attack, stroke or amaurosis fugax) and stenosis >70 % or plaques not associated with symptoms and stenosis >80 %, measured by duplex. Patients were preoperatively assessed by a neurologist. Blood samples were collected one day before endarterectomy. Informed consent was given by each patient. The study was approved by the local ethical committee. Plaques were snap-frozen in liquid nitrogen immediately after surgical removal. Plaque homogenates were prepared as previously described [18]. One mm fragments, from the most stenotic region, were taken for histology. Stains for lipids (Oil Red O), vascular smooth muscle cells (α-actin) and macrophages (CD68) were performed as previously described [19]. Measurements of the area of plaque (% area) for the different stainings were quantified blindly using BiopixiQ 2.1.8 (Gothenburg, Sweden) after scanning with ScanScope Console Version 8.2 (LRI imaging AB, Vista CA, USA).

Finally aliquots of 50 μL of plaque homogenate were centrifuged at 13,000 g for 10 min. Twenty-five μL of the supernatant was removed and used for measuring different cytokines and growth factors. The procedure was performed according to the manufacturer’s instructions (Human Cytokine/chemokine immunoassay, Millipore Corporation, MA, USA) and analyzed with Luminex 100 IS 2.3 (Austin, Texas, USA). Elastin and collagen in plaque homogenates were measured using the Fastin Elastin and Sircol soluble Collagen assays as previously described [19]. Renin levels in plaque homogenates were analyzed using the Proximity Extension Assay (see below).

### Analysis of renin and IL-6 in plasma

Plasma levels of renin and IL-6 were analyzed by the Proximity Extension Assay (PEA) technique using the Proseek Multiplex CVD^96×96^ reagents kit (Olink Bioscience, Uppsala, Sweden) at the Clinical Biomarkers Facility, Science for Life Laboratory, Uppsala. Oligonucleotide-labeled antibody probe pairs were allowed to bind to their respective targets present in the plasma sample and addition of a DNA polymerase led to an extension and joining of the two oligonucleotides and formation of a PCR template. Universal primers were used to pre-amplify the DNA templates in parallel. Finally, the individual DNA sequences were detected and quantified using specific primers by microfluidic real-time quantitative PCR chip (96.96, Dynamic Array IFC, Fluidigm Biomark). The chip was run with a Biomark HD instrument. The mean coefficients of variance for intra-assay variation and inter-assay variation are 7 and 13 % for renin, and 8 and 10 % for IL-6, respectively. All samples were analyzed in the same run. Data analysis was performed by a preprocessing normalization procedure using Olink Wizard for GenEx (Multid Analyses, Sweden). All data are presented as arbitrary units (AU). General calibrator curves to calculate the approximate concentrations as well as technical information about the assays are available on the Olink homepage (http://www.olink.com).

### Statistics

Statistical analyses were performed based on log2-transformed renin levels to approximate normal distribution. Assessment of association with other markers was done via Pearson correlation coefficient and linear regression models adjusted to study center and the individual factors from the Framingham risk score (age, gender, total cholesterol, HDL cholesterol, systolic blood pressure, and smoking). Statistical significance of the association in the linear regression model is judged by the *p*-value of the renin coefficient.

For assessing associations of renin levels with CVD risk logistic regression models were used, and adjustment for factors of the Framingham risk score and study center were done. Spearman correlations were used to determine associations between plasma renin levels and components of endarterectomy specimens. Statistical analyses of the SUMMIT study were done using the R version 3.1.1 and the IBM SPSS (version 22) software was used for the carotid endarterectomy study.

## Results

### Plasma renin levels are increased in both T2D and prevalent CVD

**Fig. 1:**
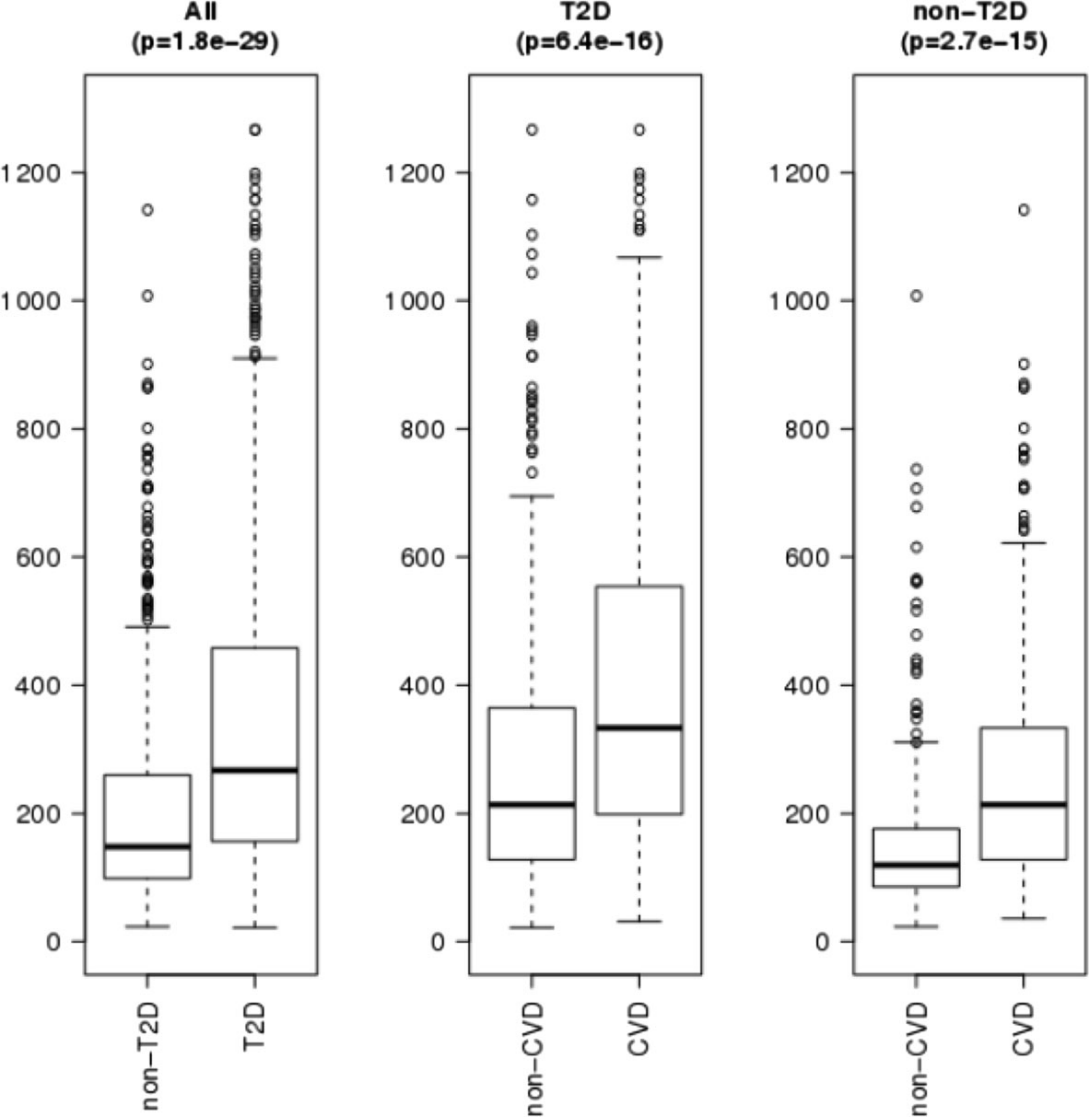
Renin plasma levels in subjects with and without T2D and with and without CVD. Values are given as arbitrary units (linear scale). *P*-values were calculated using the Mann-Whitney U test

The design of the SUMMIT study has previously been reported [17]. Briefly, this cross-sectional cohort involves 985 T2D subjects and 515 subjects without T2D matched for age, gender and prevalence of CVD recruited at four different European academic health centers (Malmö, Dundee, Exeter and Pisa). The clinical characteristics of the study cohort are shown in Table 1. Plasma renin levels were significantly higher in subjects with T2D (median (IQR) 267 (157–458) versus 148 (98–260) AU in non-T2D). Subjects with prevalent CVD had increased levels of renin in both the T2D (333 (199–554) versus 214 (128–365) AU and non-T2D groups (214 (128–333) versus 119 (87–176) AU, Fig. 1). To determine if the renin association with CVD was independent of major cardiovascular risk factors we adjusted for the factors included in the Framingham risk score (e.g. age, gender, total cholesterol, HDL, systolic blood pressure and smoking) using logistic regression models. The results demonstrated that high levels of renin were independently associated with higher odds ratios for CVD in both the T2D and non-T2D groups. The associations with CVD remained significant when adjusting for renal function (estimated glomerular filtration rate; eGFR) and in the T2D also when adjusting for treatment with RAAS inhibitors (Table 2).

**Table 1:** Clinical characteristics of the study cohort

	T2D with CVD	T2D without CVD	Non-T2D without CVD	Non-T2D with CVD
*N*	458	527	270	245
Male sex, *n*(%)	331 (72)	324 (61)	163 (60)	161 (66)
Age, years	69.3 (7.8)	66.2 (8.8)	66.4 (9.1)	67.9 (9.2)
T2D Duration, years	12.2 (8.8)	9.1 (6.9)	–	–
BMI, kg/m^2^	30.1 (4.9)	30.8 (5.5)	27.0 (4.0)	27.6 (4.1)
Medication				
Statin use, *n*(%)	401 (88)	321 (61)	97 (36)	171 (70)
Anti-hypertensive treatment use, *n*(%)	419 (92)	352 (67)	129 (48)	172 (70)
Blood pressure				
SBP, mmHg	138 (20.0)	136 (17.6)	132 (17.2)	134 (16.0)
DBP, mmHg	76 (10.3)	78 (9.8)	77 (9.4)	77 (9.2)
Pulse pressure, mmHg	62.6 (16.2)	58.2 (13.1)	54.5 (12.5)	57.2 (14.2)
Metabolic factors				
HbA1c, mmol/mol %	58.8 (13.9) 8.0 (5.0)	56.6 (13.0) 7.9 (10.0)	38.4 (3.9) 5.8 (2.3)	39.8 (4.7) 5.8 (0.5)
Tot Cholesterol, mmol/l	3.92 (0.9)	4.38 (1.0)	5.03 (1.1)	4.47 (1.1)
HDL, mmol/l	1.19 (0.35)	1.32 (0.38)	1.51 (0.44)	1.42 (0.40)
LDL, mmol/l	2.06 (0.74)	2.42 (0.91)	3.01 (0.94)	2.52 (0.88)
Triglycerides, mmol/l	1.68 (0.94)	1.62 (0.92)	1.30 (0.65)	1.37 (0.70)
Renal function				
Creatinine, serum, μmol/l	93.4 (32.2)	80.0 (20.6)	81.3 (18.5)	83.3 (19.3)
ACR, mg/mmol	9.48 (37.4)	4.14 (16.5)	3.23 (22.7)	3.38 (15.3)
eGFR	60.8 (20.3)	71.3 (19.4)	69.6 (17.7)	66.9 (18.9)

Binary variables are reported as *n*(%) and quantitative data are reported as mean (SD)

*BMI* body mass index, *SBP* systolic blood pressure, *DBP* diastolic blood pressure, *HDL* high-density lipoprotein, *LDL* low-density lipoprotein, *ACR* albumin creatinine ratio, *eGFR* estimated glomerular filtration rate

**Table 2:** Odds-ratios for CVD risk with increased plasma renin levels (log2 scale)

Covariates	All			T2D			Non-T2D		
	OR	(95 % CI)	*p*-value	OR	(95 % CI)	*p*-value	OR	(95 % CI)	*p*-value
Framingham (age, gender, total cholesterol, HDL, SBP, smoking)	1.37	(1.22–1.55)	9.5E-08	1.55	(1.34–1.80)	7.7E-09	1.45	(1.14–1.85)	3.0E-03
Framingham + eGFR	1.29	(1.14–1.45)	5.0E-05	1.42	(1.21–1.66)	1.2E-05	1.43	(1.12–1.83)	4.0E-03
Framingham + RAAS inhibitors	1.20	(1.06–1.36)	5.0E-03	1.41	(1.21–1.65)	1.3E-05	1.10	(0.84–1.44)	ns

*P*-values from separate logistic regression models adjusted for the individual components of the Framingham risk score and study center, and in addition for eGFR or RAAS inhibitors

*SBP* systolic blood pressure, *HDL* high-density lipoprotein, *eGFR* estimated glomerular filtration rate

### Association between plasma renin and risk factors

The levels of renin in plasma increased with age, body mass index (BMI), HbA1c and triglycerides, and showed inverse associations with LDL cholesterol, HDL cholesterol and renal function as assessed by the eGFR. Notably, these associations were at least as strong in non-T2D subjects as in the T2D group (Table 3). There was also a significant association with inflammation as assessed by plasma IL-6 levels in both groups. An inverse association between renin and systolic blood pressure was observed in the T2D group but not in the non-T2D group.

**Table 3:** Pearson Correlations between plasma renin levels and factors related to CVD

	T2D		Non-T2D	
	*r*	*p*	*r*	*p*
Age	0.16 (0.10–0.23)	4.2E-07	0.26 (0.18–0.34)	2.5E-09
BMI	0.08 (0.01–0.14)	1.6E-02	0.19 (0.10–0.27)	2.0E-05
HbA1c	0.20 (0.14–0.26)	7.4E-10	0.25 (0.16–0.33)	3.3E-08
Systolic BP	−0.15 (−0.21–0.09)	4.0E-06	−0.04 (−0.13–0.05)	ns
HDL chol	−0.15 (−0.21–0.09)	4.8E-06	−0.21 (−0.29–0.12)	3.8E-06
LDL chol	−0.20 (−0.27–0.14)	8.4E-10	−0.28 (−0.36–0.19)	1.2E-09
Triglycerides	0.12 (0.06–0.19)	1.8E-04	0.09 (0.00–0.18)	5.0E-02
eGFR	−0.29 (−0.35–0.23)	<1.0E-16	−0.24 (−0.32–0.16)	6.9E-08
Plasma IL6	0.28 (0.22–0.33)	<1.0E-16	0.29 (0.21–0.37)	4.9E-11

*BMI* body mass index, *SBP* systolic blood pressure, *HDL* high-density lipoprotein, *LDL* low-density lipoprotein, *eGFR* estimated glomerular filtration rate

### Association between plasma renin and measures of atherosclerosis, arterial stiffness and endothelial function

We next analyzed if renin levels were related to the severity of vascular changes. Peripheral artery disease was determined by the ankle-brachial pressure index (ABPI), carotid disease by the intima-media thickness (IMT) in the common carotid artery (CCA) and the carotid bulb as well as by carotid plaque area, arterial stiffness by measuring pulse wave velocity (PWV) and endothelial function by determining the Reactivity Hyperemia Index (RHI). Subjects with T2D had more severe carotid disease, increased arterial stiffness, and impaired endothelial function while there was no difference in ABPI between subjects with and without T2D (Table 4). Renin levels were significantly associated with the severity of atherosclerosis both in peripheral arteries and in the carotids. These associations were similar in subjects with and without T2D. Moreover, with the exception of the association between renin and CCA IMT in the non-T2D group they remained significant when adjusting for age, gender, total cholesterol, HDL, smoking and importantly also for systolic blood pressure (Table 5). There was a weak risk factor-independent relationship between high renin levels and a lower RHI among T2D subjects, but otherwise we found no independent associations with arterial stiffness or endothelial dysfunction (Table 5).

**Table 4:** Differences in vascular changes between subjects with and without T2D

	T2D	Non-T2D	*p*-value
ABPI	1.16 (1.07, 1.25)	1.17 (1.08, 1.25)	ns
ABPI (right)	1.16 (1.07, 1.25)	1.17 (1.09, 1.25)	ns
ABPI (left)	1.16 (1.07, 1.25)	1.17 (1.08, 1.25)	ns
CCA IMT	0.91 (0.80, 1.04)	0.85 (0.75, 0.99)	5.1E-06
CCA IMT (right)	0.89 (0.77, 1.03)	0.84 (0.74, 0.97)	4.9E-05
CCA IMT (left)	0.91 (0.78, 1.06)	0.86 (0.74, 1.02)	5.9E-05
Bulb IMT	1.11 (0.93, 1.42)	1.05 (0.90, 1.30)	1.0E-03
Bulb IMT (right)	1.08 (0.90, 1.41)	1.01 (0.87, 1.30)	1.6E-03
Bulb IMT (left)	1.09 (0.92, 1.37)	1.05 (0.89, 1.28)	0.01
Plaque area	17.0 (10.7, 26.1)	15.6 (10.4, 22.6)	0.01
PWV	10.9 (9.3, 13.2)	9.6 (8.3, 11.4)	2.0E-16
RHI	2.01 (1.72, 2.48)	2.31 (1.90, 2.74)	4.1E-11

For each group the median is provided with the interquartile range, *p*-values from Mann-Whitney U-test

*ns* non significant, *ABPI* ankle brachial pressure index, *CCA* common carotid artery, *IMT* intima media thickness, *PWV* pulse wave velocity, *RHI* reactive hyperemia index

**Table 5:** Association between plasma renin levels and measures of atherosclerosis burden, arterial stiffness and endothelial function

		T2D	Non-T2D
ABPI	*r*	−0.19	−0.15
	95 % CI	(−0.26, −0.13)	(−0.23, −0.06)
	pCor	7.7E-09	0.001
	r2	0.14	0.20
	pReg	7.4E-09	0.006
CCA IMT	*r*	0.15	0.19
	95 % CI	(0.08, 0.21)	(0.10, 0.27)
	pCor	1.2E-05	4.7E-05
	r2	0.15	0.29
	pReg	0.015	ns
Bulb IMT	*r*	0.27	0.30
	95 % CI	(0.20, 0.33)	(0.21, 0.38)
	pCor	1.1E-14	1.8E-10
	r2	0.22	0.28
	pReg	8.7E-07	0.001
Plaque area	*r*	0.25	0.27
	95 % CI	(0.18, 0.32)	(0.17, 0.36)
	pCor	6.4E-12	1.4E-07
	r2	0.25	0.31
	pReg	3.3E-09	3.70E-05
PWV	*r*	0.08	0.18
	95 % CI	(0.01, 0.15)	(0.09, 0.27)
	pCor	0.029	1.2E-04
	r2	0.33	0.36
	pReg	ns	ns
RHI	*r*	−0.14	−0.14
	95 % CI	(−0.21, −0.08)	(−0.23, −0.05)
	pCor	1.8E-05	0.002
	r2	0.08	0.15
	pReg	0.025	ns

*r* Pearson correlation coefficient, *95 % CI* 95 % confidence interval for r, *pCor p*-value for correlation significance, *r2* proportion of variation explained by linear regression model adjusted for age, gender, total cholesterol, *HDL* systolic blood pressure, smoking, and study center, *pReg p*-value for significance of renin coefficient in this model; data of imaging variables has been log2-transformed to resemble normal distribution, *ABPI* ankle brachial pressure index, *CCA* common carotid artery, *IMT* intima media thickness, *PWV* pulse wave velocity, *RHI* reactive hyperemia index

### Influence of RAAS inhibition

In an additional approach to explore the role of RAAS activation in vascular complications to T2D we analyzed if ongoing treatment with RAAS inhibitors (e.g. ACE inhibitors ARBs or renin inhibitors) was associated with any difference in markers of atherosclerosis, arterial stiffness and endothelial dysfunction. Subjects with or without prevalent CVD were analyzed separately since prevalent CVD has been shown to be associated with more severe vascular pathologies [17]. Seventy-seven percent of T2D subjects with prevalent CVD and 57 % of the group with T2D subjects without prevalent CVD received treatment with any type of RAAS-inhibitor. Increased renin levels were observed in subjects treated with RAAS-inhibitors both in the CVD (median (IQR) 355 (231–584) versus 224 (151–377) AU) and non-CVD groups (271 (167–474) versus 167 (108–254) AU). No significant differences in ABPI, carotid IMT, carotid plaque area, PWV or RHI between T2D subjects with or without RAAS inhibitors (data not shown). In the non T2D subjects RAAS-inhibition was associated with increased plaque area in the CVD group and increased CCA IMT, carotid bulb IMT and PWV in the non-CVD group (Table 6).

**Table 6:** Differences in vascular changes in non-T2D subjects without or without treatment with any type of RAAS inhibitor

	No T2D with CVD					No T2D without CVD				
Variable	Yes	No	MedianYes	MedianNo	*p*-value	Yes	No	MedianYes	MedianNo	*p*-value
ABPI	146	76	1.16 (1.08–1.25)	1.13 (1.02–1.23)	ns	49	196	1.17 (1.12–1.23)	1.17 (1.10–1.25)	ns
CCA IMT (mm)	147	73	0.88 (0.78–1.04)	0.88 (0.78–0.99)	ns	48	192	0.92 (0.82–1.03)	0.81 (0.72–0.96)	7.4E-04
Bulb IMT (mm)	132	66	1.20 (0.98–1.44)	1.07 (0.96–1.31)	ns	47	187	1.15 (0.99–1.38)	0.96 (0.84–1.15)	1.9E-04
Plaque area (mm^2^)	128	67	18.5 (12.5–24.6)	13.58 (10.9–18.7)	0.004	40	134	15.8 (10.7–29.8)	13.2 (7.8–20.0)	ns
PWV (m/sec)	135	63	10.2 (8.3–11.8)	9.7 (8.5–11.8)	ns	46	189	9.8 (9.0–11.0)	9.2 (7.9–10.7)	0.025
RHI	158	77	2.2 (1.8–2.6)	2.3 (1.9–2.7)	ns	50	196	2.25 (1.88–2.48)	2.41 (2.00–2.81)	ns

Difference of imaging marker was assessed by Mann-Whitney U-test, medians are presented together with IQRs

*ns* not significant, *ABPI* ankle brachial pressure index, *CCA* common carotid artery, *IMT* intima media thickness, *PWV* pulse wave velocity, *RHI* reactive hyperemia index

To explore the possibility that the renin associations with CVD and atherosclerosis burden was explained by a more frequent treatment with RAAS-inhibitors in subjects with more advanced disease we next analyzed subjects with and without RAAS-inhibitors separately. Subjects treated with renin inhibitors (*n* = 68) were excluded from these analyses as these do not have the same increasing effect on plasma renin as ACE inhibitors and ARBs. Moreover, since only 41 subjects in the non-CVD, non-T2D group were treated with ACE inhibitors and ARBs we restricted the analyses to the T2D group. In T2D subjects treated with RAAS inhibitors those with CVD (*n* = 313) had higher plasma renin than those without CVD (*n* = 256; 355 (231–584) versus 271 AU (167–465), *p* = 9.2E-06). Also in T2D subjects without RAAS inhibition higher renin levels was observed in the CVD group (*n* = 150) than in the non-CVD group (*n* = 211; 224 (151–377) versus 167 AU (108–253), *p* = 3.3E-05). The odds-ratio for CVD risk with increased plasma renin levels was 1.45 (95 % C.I. 1.19–1.76) for T2D subjects treated with RAAS inhibitors and 1.41 (95 % C.I. 1.04–1.93) for those not treated with RAAS inhibitors when controlling for age, gender, total cholesterol, HDL, systolic blood pressure, smoking and study center. The pattern of renin associations with measures of atherosclerosis burden, arterial stiffness and endothelial function were also similar in T2D subjects with or without treatment with RAAS inhibitors (Table 7). Taken together, these observations demonstrate that the renin associations with CVD and atherosclerosis burden observed in the present study is not due a more frequent treatment with RAAS-inhibitors in subjects with more advanced disease.

**Table 7:** Associations between renin levels and measures of atherosclerosis burden, arterial stiffness and endothelial function

		T2D	
		RAASi	NoRAASi
ABPI	*r*	−0.19	−0.09
	pCor	1.6E-05	ns
	r2	0.14	0.16
	pReg	3.6E-04	ns
CCA IMT	*r*	0.14	0.13
	pCor	7.9E-04	0.021
	r2	0.15	0.20
	pReg	0.007	ns
Bulb IMT	*r*	0.26	0.29
	pCor	1.0E-08	9.40E-07
	r2	0.20	0.3
	pReg	3.3E-05	0.029
Plaque area	*r*	0.26	0.20
	pCor	7.7E-09	0.002
	r2	0.26	0.29
	pReg	3.2E-08	ns
PWV	*r*	0.02	0.09
	pCor	ns	ns
	r2	0.31	0.38
	pReg	ns	ns
RHI	*r*	−0.16	−0.15
	pCor	2.3E-04	0.007
	r2	0.09	0.11
	pReg	0.036	ns

*r* is Pearson correlation coefficient, and r2 is proportion of variation explained by a linear regression model adjusted for age, gender, total cholesterol

*HDL* systolic BP, smoking, eGFR, IL6, and study center

*RAASi* RAAS inhibitor treatment

### Plasma renin levels and atherosclerotic plaque structure

Since previous experimental data have shown that RAAS activation can stimulate inflammation, expression of fibrous proteins and smooth muscle cell growth we investigated if plasma levels of renin were related to atherosclerotic plaque content of these factors in a cohort of 205 carotid endarterectomy patients. The clinical characteristics of this cohort have been reported previously [19]. Weak but statistically significant associations were found between plasma renin levels and the plaque content of platelet-derived growth factor (*r* = 0.14, *p* < 0.05) and TNF-α (*r* = −0.16, *p* < 0.05), but there were no significant associations with plaque content of collagen, elastin, IL-6, monocyte chemotactic protein-1, vascular endothelial growth factor or immunostaining for macrophages or smooth muscle cells (data not shown). Renin levels in plaque homogenates were analyzed using the Proximity Extension Assay. There was a strong correlation between the levels of renin in plasma and atherosclerotic plaque tissue (*r* = 0.80, *p* = 1.0E-40). However, with the exception of a trend for a correlation with the plaque content of platelet-derived growth factor (*r* = 0.13, *p* = 0.07) there were correlations between the plaque renin content and other plaque components.

## Discussion

There is evidence from clinical trials that treatment with RAAS inhibitors slows the onset of T2D and reduces the risk of renal complications in manifest T2D [20]. Moreover, experimental studies have shown that activation of RAAS stimulates processes known to be of importance for development of atherosclerosis including inflammation, oxidative stress, smooth muscle cell growth and fibrosis suggesting that it could play an important role also in the macrovascular complications associated with T2D [21]. The concept that RAAS promotes atherogenesis in diabetes has gained support from a few animal studies [11, 12], but evidence from randomized clinical trials of RAAS inhibitors with cardiovascular end points has been inconsistent [22]. Thus, the clinical importance of RAAS activation in the development of cardiovascular complications in T2D remains to be fully established. In the present study we investigated if RAAS activation, as assessed by circulating renin levels, was associated with the severity of vascular complications in T2D subjects with and without prevalent CVD. Our findings show that there is a significant association between circulating renin levels and atherosclerotic burden in the carotid and peripheral arteries in subjects with T2D and that these associations are independent of systolic blood pressure and other major cardiovascular risk factors. In accordance, T2D subjects with clinically manifest CVD were characterized by increased renin levels and this difference remained significant when adjusting for renal function, treatment with RAAS-inhibitors and cardiovascular risk factors including systolic blood pressure. Collectively these observations are well in line with the proposed role for RAAS activation in cardiovascular complications in T2D. Notably, plasma renin levels were elevated also in non-T2D subjects with prevalent CVD and demonstrated significant associations with markers of atherosclerosis also in this group. Moreover, renin levels were found to link with factors characteristic for T2D, such as HBA1c, BMI and low HDL, also among subjects without T2D. However, renin levels were lower in subjects without T2D and the vascular changes were less pronounced. These observations are in line with previous findings that insulin resistance stimulates the expression of renin and that this may contribute to a more severe progression of atherosclerosis in T2D [1, 2, 5].

In spite of the association between renin and markers of atherosclerotic burden we found no evidence for reduced atherosclerosis in subjects treated with RAAS-inhibitors. There are several possible explanations to this finding. First, it cannot be excluded that RAAS activation has no direct effect on atherosclerosis development in humans and that the association between renin and disease severity observed here is caused by association of renin with another atherogenic factor. However, the fact that renin remained significantly associated to atherosclerotic burden when adjusting for other cardiovascular risk factors, as well as renal function argues against this. Second, it could be that vascular changes caused by increased RAAS activation over many years are not easily reversible by providing RAAS inhibition in different time-frames. Third, it is possible that RAAS inhibition when given alone may be sufficient to reduce or inhibit the progression of disease but when co-administered with other potent anti-atherogenic drugs, such as statins, the effect of RAAS inhibition may become too small to be of clinical significance. In line with the latter possibility treatment with ACE-inhibition reduced myocardial events in the HOPE study in which only about 20 % received lipid-lowering therapy [14] while no significant effect of ACE-inhibition on major coronary events could be observed in the ADVANCE trial in which about 50 % received lipid-lowering therapy [23]. Unexpectedly, RAAS-inhibition was associated with more severe carotid atherosclerosis in non-T2D subjects. The reason for this association remains to be elucidated but it does not support the existence of an anti-atherogenic effect of RAAS inhibitors.

Experimental studies have shown that angiotensin II induces vascular oxidative stress leading to decreased endothelial relaxation and endothelial dysfunction [24]. In accordance with these findings we observed an inverse correlation between plasma renin and the RHI in the present study. However, no differences were observed in RHI between subjects with or without RAAS-inhibition. Angiotensin II has also been shown to affect processes involved in atherosclerotic plaque development and stability. Increased expression of adhesion molecules and pro-inflammatory cytokines including monocyte chemotactic protein-1 and IL-6 has been reported in monocytes, smooth muscle cells and endothelial cells exposed to angiotensin II [25–28]. Additionally, angiotensin II has been shown to stimulate smooth muscle cell growth and collagen production [10, 29, 30]. In the present study we found no or only weak associations between plasma renin and the expression of pro-inflammatory cytokines, fibrous proteins and smooth muscle cells in atherosclerotic plaques obtained from carotid endarterectomy patients. Taken together these findings suggest that RAAS activation does not significantly affect plaque composition and vulnerability. However, since the experimental data show that angiotensin II stimulates both inflammatory and repair processes it is possible that it may contribute to plaque development without affecting the balance between individual plaque components. It is also possible that local RAAS activation is of more importance for these processes than systemic RAAS activation.

There are some limitations of the present study that should be considered. Most importantly, the observational design of the study does not allow for conclusions regarding cause and effect relations. Thus, our findings do not provide clinical evidence for an atherogenic role of renin and RAAS activation. They are, however, well in line with experimental data suggesting that RAAS activation exuberate several biological processes involved in plaque development. The finding that treatment with RAAS-inhibitors does not affect atherosclerosis severity also needs to be interpreted with caution since this is an observational study and information regarding length of treatment is unfortunately lacking. Another important limitation is that RAAS activation was only assessed indirectly through analysis of renin levels.

## Conclusions

Our findings provide clinical evidence for an association between RAAS activation and atherosclerotic burden both in subjects with and without T2D. Plasma renin and atherosclerosis are concomitantly increased in subjects with T2D as compared to age and sex-matched subjects without T2D suggesting that these associations may be of particular importance in vascular complication of diabetes. Importantly, the association between renin and atherosclerotic burden was independent of blood pressure and other major cardiovascular risk factors suggesting involvement of a direct effect of RAAS activation on the vascular wall. However, our findings also suggest that treatment with RAAS inhibitors may have limited effectiveness in counteracting the atherogenic effects of RAAS activation.

## Abbreviations

ABPI: Ankle-brachial pressure index
ACE: Angiotensin-converting enzyme
ARBs: Angiotensin receptor blockers
CVD: Cardiovascular disease
IMT: Carotid intima-media thickness
PWV: Pulse wave velocity
RAAS: Renin-angiotensin-aldosterone-system
RHI: Reactivity hyperemia index
SUMMIT: SUrrogate markers for Micro- and Macro-vascular hard endpoints for Innovative diabetes Tools
T2D: Type 2 diabetes
